# Circulating/cerebrospinal T lymphocytes as indicators of clinical prognosis in intracerebral hemorrhage: A prospective study

**DOI:** 10.1097/MD.0000000000035827

**Published:** 2024-07-19

**Authors:** Qian Xu, Shuangbo Fan, Liang Wang, Ji Zheng, Yulin Wan, Rudong Tian, Jia Xia, Zhenping Zhao

**Affiliations:** a Department of Neurosurgery, Zhenhai People’s Hospital, Ningbo, Zhejiang, China.

**Keywords:** cerebrospinal fluid, Glasgow Coma Scale, intracerebral hemorrhage, peripheral blood, T lymphocytes

## Abstract

Secondary injury of cerebral hemorrhage is induced by systemic inflammatory cascades, which are related to perihematomal brain edema, cellular apoptosis, and the disruption of the blood–brain barrier. This study was to specifically elaborate the relationship of circulating/cerebrospinal T lymphocytes and Glasgow Coma Scale (GCS) score at 6 months after intracerebral hemorrhage (ICH). The enrolled patients were divided into 2 groups based on GCS score: the favorable prognosis group (GCS > 12) and unfavorable prognosis group (GCS ≤ 12). T lymphocyte subpopulations were analyzed by flow cytometry. A total of 30 samples of peripheral blood and 17 samples of cerebrospinal fluid were collected and analyzed, including 19 cases and 12 cases in the favorable prognosis group (GCS > 12) respectively. Both CD3^+^ and CD3^+^CD4^+^ T lymphocyte counts on Day 1 after ICH were lower in the peripheral blood of patients with unfavorable prognosis (GCS ≤ 12) (*P* = .025 and .022, respectively). There were correlation trends between the GCS scores and CD3^+^ T lymphocyte count (*P* = .0144), and CD3^+^CD4^+^ T lymphocyte count (*P* = .0135). In cerebrospinal fluid, there was a close correlation between the GCS scores and CD3^+^CD4^+^ percentage, CD4^+^/CD8^+^ ratio, CD3^+^ and CD3^+^CD4^+^ T lymphocyte counts. The area under the curve of CD4^+^/CD8^+^ T lymphocyte ratio was the largest among them (*P* = .000 and area under the curve = 0.917), with a significantly high specificity and sensitivity (0.917 and 1.000). Based on cerebrospinal fluid samples, the CD4^+^/CD8^+^ T lymphocyte ratio on Day 1 after ICH may be a more significant indicator to predict the short-term prognosis at 6 months after ICH.

## 1. Introduction

Cerebral hemorrhage is a serious neurovascular disorder including 2 pathological phases: primary injury and secondary injury.^[1]^ The primary injury is mainly caused by hematoma expansion. And the secondary injury is induced by systemic inflammatory cascades, which are related to perihematomal brain edema, cellular apoptosis, and the disruption of the blood–brain barrier.^[2]^ It is well known that macrophages and immune cells are involved in secondary injury after intracerebral hemorrhage (ICH).^[3]^ It was reported that the percentage of infiltrated T lymphocytes (CD3^+^) in the ipsilateral hemisphere was increased and proinflammatory cytokines the infiltrated T lymphocytes secreted subsequently reinforced inflammation and cerebral edema.^[4]^ This immune response was also reported by Liu et al that the increment of T lymphocyte counts appeared only 6 hours after ICH in perihematomal region.^[5]^ And different from ischemic stroke, T lymphocytes are the major leukocyte population in the region of ICH.^[6]^

The former study indicated that subpopulations of lymphocytes, like CD4^+^ lymphocytes, and the ratio of CD4^+^/CD8^+^ lymphocytes in the peripheral blood may be valuable predictors of intracranial hypertension in ICH patients.^[7]^ The potential role of CD4^+^ and CD8^+^ T lymphocytes as prognostic indicator in ICH was not explored yet. To investigate the role of circulating/cerebrospinal T lymphocytes in prognostic analysis, we prospectively collected and analyzed the changes of circulating T cells on Day 1, 7 and 14 after ICH, and their relationship with Glasgow Coma Scale (GCS) scores, based on receiver operating characteristic curve of CD3^+^, CD3^+^CD4^+^ and CD4^+^/CD8^+^ ratio for GCS score.

## 2. Methods

### 2.1. Management of patient

All recruited patients were from the inpatient department of the neurosurgical care unit in Zhenhai People’s Hospital, Ningbo city. The diagnosis of cerebral hemorrhage was diagnosed by a head CT scan before enrollment. Inclusion and exclusion criteria included: (1) adult patients (age >18 years); (2) GCS score was recorded since admission within 24 hours after ICH; (3) without second craniotomy; (4) without kidney or liver failure, urinary tract infection or hematologic diseases. All patients or their guardians were fully informed to approve this trial. This clinical study was approved by the local medical ethics committee (No. 2019012).

All patients were performed with appropriate surgery, including decompressive craniectomy, hematoma removal, ventriculotomy, and drainage, cranioplasty and so on. All open surgical incisions were treated with antibiotics, and other types of incisions were not. The postoperative wound infection rate was controlled below 1%. From the onset of the disease to surgery after admission, the period of time was generally controlled within 90 minutes. GCS scores were recorded at 6 months after the enrollment of patients. The aliquot of peripheral blood sample and cerebrospinal fluid (if possible) was saved in EDTA K2 vacuum tubes (PUTH, Chengdu, China) on Day 1, 7 and 14 after ICH. The cerebrospinal fluid sample for the first time was obtained from the drainage tube placed after the operation, with a relatively pale color to avoid blood. In addition, cerebrospinal fluid samples were extracted by lumbar puncture at each time point. Basic patient information (including medical history, gender, age, GCS score, etc) was recorded and matched with the blood sample.

The systolic blood pressure was controlled under 140 mm Hg during the perioperative period for the ICH management.^[8]^ The measure of other clinical signs included temperature control, the maintenance of fluid balance and the prevention of infection.

GCS scores are commonly measured on 3 aspects: eye-opening response, verbal response and motor response. For head injury, the ICH patient was classified as conscious with GCS score of 15, mild head injury with GCS score of 13 to 14, moderate head injury with GCS score of 9 to 12, severe head injury with GCS score <8.^[9]^ Osmotic dehydration was executed with the administration of mannitol or glycerol fructose if intracerebral pressure was elevated (>20 mm Hg). The patients were then divided into 2 groups: the favorable prognosis group (GCS > 12) and unfavorable prognosis group (GCS ≤ 12). For outcome evaluation, the GCS score at 6 months after ICH was identified as the endpoint of clinical prognosis (Table 1).

**Table 1:** Demographic data of the study cohort.

Characteristic	GCS > 12 (n = 19)	GCS ≤ 12 (n = 11)	*P* value
Age (years)	58.9 ± 8.0	53.3 ± 11.6	.13
Gender, male/female	16/3	10/1	.52
Hematoma spot			.08
Cerebral hemorrhage	12 (63.2)	9 (81.8)	
Basal ganglia hemorrhage	7 (36.8)	2 (18.2)	
Surgery type, n (%)			
Intracerebral hematoma removal	16 (84.2)	9 (81.8)	.92
Middle cerebral artery aneurysm clipping	8 (42.1)	5 (45.5)	.87
Transcatheter coil embolization of intracranial aneurysm	4 (21.1)	3 (27.3)	.69
Hematoma size	36.4 ± 5.1	38.2 ± 4.8	.63
Period of time from ICH onset to surgery			
GCS scores	14.0 ± 0.88	6.6 ± 2.62	.000

GCS = Glasgow Coma Scale, ICH = intracerebral hemorrhage.

### 2.2. Flow cytometry analysis of T lymphocytes

The lymphocytes were extracted from peripheral blood or cerebrospinal fluid according to previous literature.^[10]^ The samples were mixed slowly with PBS and lymphocyte separation medium. The mixture was centrifuged at 1500 rpm at 4 °C for 10 minutes. The buffy coat was then extracted and shifted into another centrifuge tube, subsequently resuspended with 500 mL of PBS. After centrifugation under the same condition, the supernatant was discarded, and cells were washed twice by PBS.

The 20 μL dilution buffers of antibodies CD3, CD4, and CD8 (eBioscience, San Diego, CA) were added into the resuspended cells to incubate under a low speed vortex for 0.5 hour at room temperature. Then 5 μL working buffer of mitochondrial staining solution (Mito-Tracker Deep Red: Ex = 644 nm, Em = 665 nm; labeled as Mito-) was added to incubate for another 1 hour. After the above steps, 500 μL of PBS was added to the tube to wash lymphocytes 3 times. Besides, 50 μL heparin and 450 μL of 1× hemolysin were placed in a flow tube and incubated at room temperature for 15 minutes in the dark. The final samples were preserved in PBS for a flow cytometry assay within 12 hours.

### 2.3. Statistical analysis

Multivariate data analyses were performed with GraphPad Prism 7 (GraphPad software Inc., San Diego, CA). The significance of differences in gender or hematoma spot was analyzed using the χ^2^ test. The Student *t* test was used to assess the significance of the differences in CD3^+^, CD4^+^, and CD8^+^ T cells between 2 groups. Pairwise comparison of the area under the curve (AUC) was analyzed using the z test. The quantified data were presented as mean ± SD, with significant differences identified as *P* < .05.

## 3. Results

### 3.1. Patient enrollment

A total of 30 ICH patients were enrolled between December 2019 and December 2020 according to our inclusion criteria. Demographic data for the study cohort were shown in Table 1. Nineteen of these 30 cases were enrolled in the favorable prognosis group (GCS > 12), and 11 cases were enrolled in the unfavorable prognosis group (GCS ≤ 12). There were no significant differences between the 2 groups in terms of age, sex, and hematoma spot. However, the GCS scores were significantly different between the 2 groups (*P* = .000).

### 3.2. Absolute count of T lymphocyte subsets (CD3^+^, CD3^+^CD4^+^) in peripheral blood as prognostic indicators

**Figure 1.**
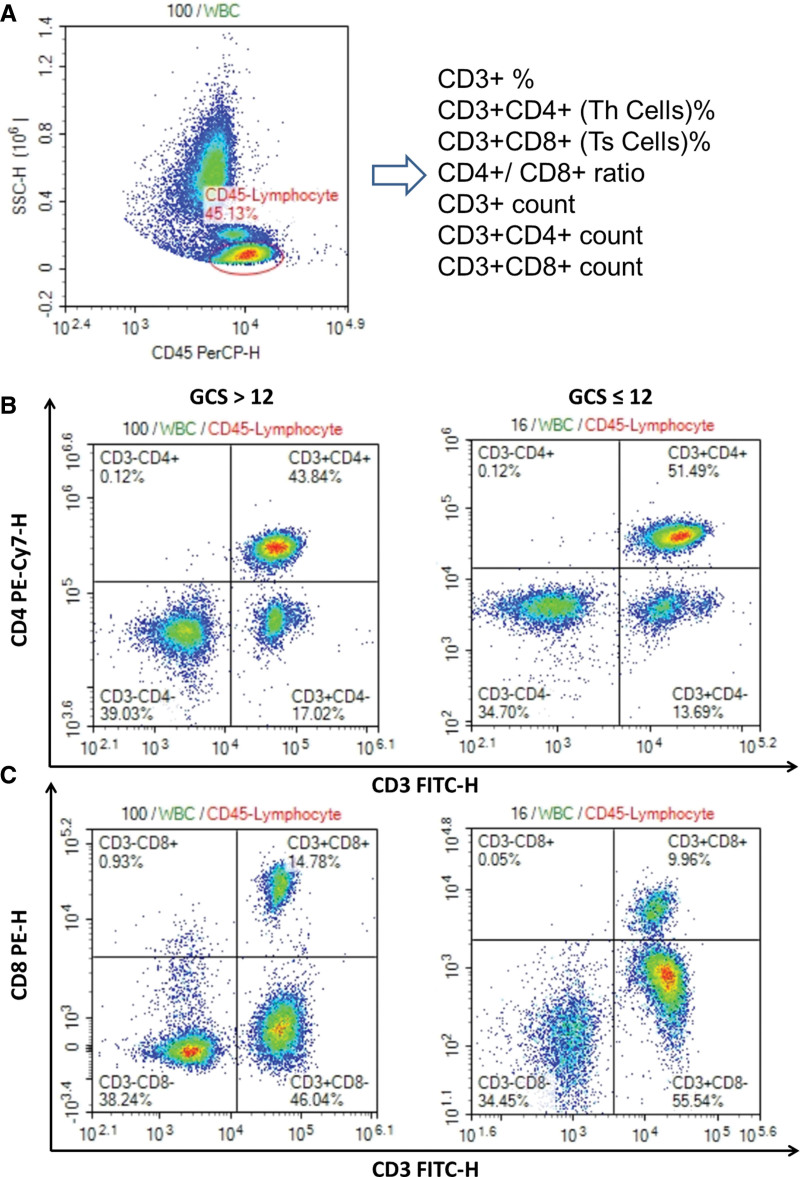
The information of T lymphocyte subsets was classified accordingly.

Blood samples were collected on Day 1, 7 and 14 after ICH. Many T lymphocyte subsets were tested by flow cytometry, and the data was classified into the favorable prognosis group (GCS > 12) and the unfavorable prognosis group (GCS ≤ 12) as shown in Figure 1. There was no difference in the different time-point of samples between the 2 groups, except absolute counts of CD3^+^ and CD3^+^CD4^+^ lymphocytes. The absolute counts of CD3^+^ lymphocytes and CD3^+^CD4^+^ T lymphocytes on Day 1 after ICH were obviously lower in the favorable prognosis group (GCS > 12), compared to the unfavorable prognosis group (GCS ≤ 12) (*P* = .025 and .022, respectively) (see Table S1, Supplemental Digital Content, http://links.lww.com/MD/N224, which illustrated the characteristics of circulating T lymphocytes in the study cohort). The significant changes of T lymphocyte subsets were mainly shown at the beginning of ICH onset.

**Figure 2.**
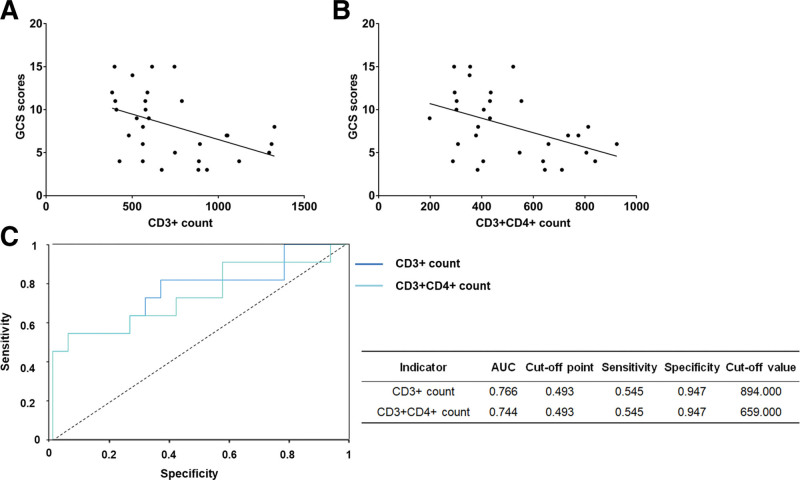
Positive correlations between GCS score and CD3^+^ lymphocyte count (A), CD3^+^CD4^+^ lymphocyte count (B), and area under the curve of indicators (C).

In Table S2, Supplemental Digital Content, http://links.lww.com/MD/N225, the AUC showed that the counts of circulating CD3^+^ lymphocytes, CD3^+^CD4^+^ T lymphocytes were valuable indicators as potential prognostic indicators (see Table S2, Supplemental Digital Content, http://links.lww.com/MD/N225, which illustrated the AUC value of circulating T lymphocytes in the study cohort for GCS score). We found positive correlations between GCS score and CD3^+^ lymphocyte count (R^2^ = 0.196, *P* = .0144) as well as between GCS score and CD3^+^CD4^+^ lymphocyte count (R^2^ = 0.199, *P* = .0135) in Figure 2A and B. Their AUC values were close to 0.766 and 0.744, with the same value of sensitivity and specificity (0.545 and 0.947, respectively) in Figure 2C.

### 3.3. Potential relationship between T lymphocyte and GCS score in cerebrospinal fluid

Cerebrospinal fluid samples were collected on Day 1, 7 and 14 after ICH. The absolute counts of CD3^+^CD4^+^ and CD3^+^CD8^+^ T lymphocytes on Day 1 after ICH were obviously lower in the favorable prognosis group (GCS > 12), compared to the unfavorable prognosis group (GCS ≤ 12) (*P* = .022 and .046, respectively) (see Table S3, Supplemental Digital Content, http://links.lww.com/MD/N226, which illustrated the characteristics of cerebrospinal T lymphocytes in the study cohort).

**Figure 3.**
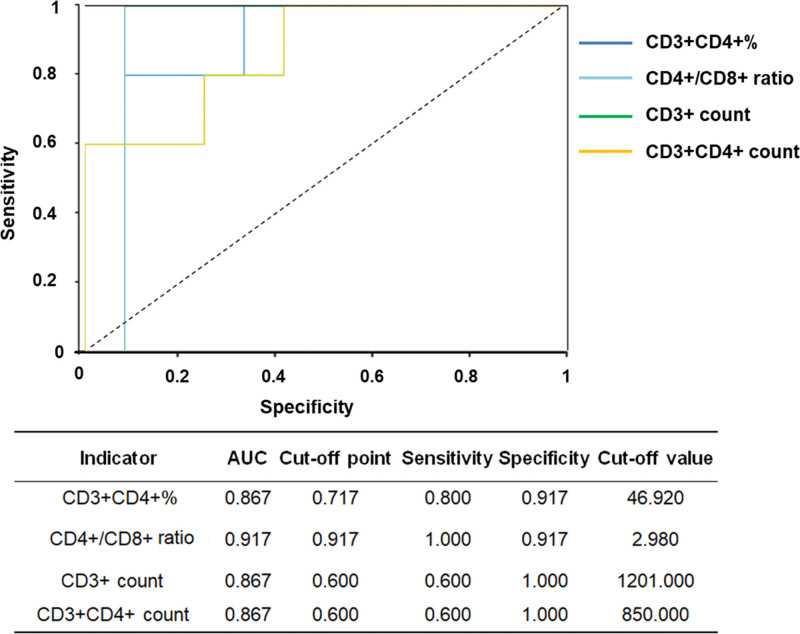
Receiver operating characteristic curve of T lymphocyte subsets for GCS score.

In the subsequent relationship study of indicators, the AUC values showed that only the percentage of CD3^+^CD4^+^ lymphocytes, CD4^+^/CD8^+^ ratio, absolute counts of CD3^+^ and CD3^+^CD4^+^ lymphocytes were valuable indicators as potential prognostic indicators (see Table S4, Supplemental Digital Content, http://links.lww.com/MD/N227, which illustrated the AUC value of cerebrospinal T lymphocytes in the study cohort for GCS score). It was indicated that the significant changes of T lymphocyte subsets at the beginning of ICH onset were more important for the final prognosis. According to their AUC values, the CD4^+^/CD8^+^ ratio was the most important indicator with the highest AUC value of 0.917, followed by sensitivity and specificity of 1.0 and 0.917, respectively in Figure 3. CD4^+^ and CD8^+^ T lymphocytes are known as positive and negative action in the immune response; therefore, the CD4^+^/CD8^+^ ratio represents the immune balance. The potential relationship between the CD4^+^/CD8^+^ ratio in cerebrospinal fluid and GCS reflects the relative immune balance and its response to the endpoint of clinical prognosis.

## 4. Discussion

In this study, we investigated the relationship of circulating/cerebrospinal T lymphocytes and GCS score at 6 months after ICH. We found that: (1) in the peripheral blood, the absolute counts of CD3^+^ lymphocytes and CD3^+^CD4^+^ T lymphocytes on Day 1 after ICH were obviously lower in the favorable prognosis group (GCS > 12), compared to the unfavorable prognosis group (GCS ≤ 12) (*P* = .025 and .022, respectively). There were positive correlations between GCS score and CD3^+^ lymphocyte count as well as between GCS score and CD3^+^CD4^+^ lymphocyte count. Based on peripheral blood samples of ICH patients, both CD3^+^ and CD3^+^CD4^+^ T lymphocyte count on Day 1 after ICH could be used as prognostic indicators for their good correlation trends. (2) In cerebrospinal fluid, according to their AUC values, the CD4^+^/CD8^+^ ratio was the most important indicator with the highest AUC value of 0.917, followed by sensitivity and specificity of 1.0 and 0.917, respectively. Among these correlations, the CD4^+^/CD8^+^ T lymphocyte ratio in cerebrospinal fluid was the best indicator of ICH.

We have learnt from previous studies that the inflammatory pathway and oxidative stress are involved in secondary brain injury after ICH to a large extent, and that the inflammatory indicators are mainly mediated by lymphocytes.^[11–13]^ According to our flow cytometer analysis, T lymphocyte subsets constituted the main immunologic response in closed head injury, as they infiltrated into the lesions contributing to early edema, which is identical with the former study about correlation of circulating T lymphocytes and intracranial hypertension in ICH.^[7]^ The similar situation of T lymphocytes was also reported in cerebral tissue of ischemic stroke in 1 meta-analysis that circulating T cell counts decreased gradually after ICH.^[14]^

To the best of our knowledge, this prospective study revealed a critical association between circulating/cerebrospinal T cells at early stage (Day 1, 7, and 14 after ICH) and GCS score at 6 months as the endpoint of clinical prognosis. We hypothesized that the CD4^+^/CD8^+^ ratio in cerebrospinal fluid was the most important indicator with the highest AUC value. To study and confirm this correlation would promote the development of new strategies against ICH.

There are many limitations for this study as well. First of all, the sample size included in this study was limited by the funding support, especially the accuracy of results based on spinal samples might be influenced by the small dataset. It should be clearly underlined that our study was preliminary and hypothesis generating. We may enroll more ICH patients in further clinical projects. Secondly, we also actually tried to evaluate the mitochondrial function and damage index in circulating/cerebrospinal samples as well.^[15,16]^ However, the change of mitochondrial function was not associated with our endpoint, GCS score. Thus, the related information was not included in this study, as the clinical evidence of this negative result was not strong enough as the sample size was small. Finally the crosstalk between microglia, T cells and nature killer cells may also be studied in further study,^[17–19]^ which may be a more systematic evaluation of the immune system and its role in clinical prognosis of ICH patients.

## 5. Conclusion

Totally, all evidence testified that T lymphocytes were strongly associated with the progress of ICH patients. The CD4^+^/CD8^+^ ratio in cerebrospinal fluid may be a valuable indicator for predicting postoperative prognosis after ICH.

## Author contributions

**Conceptualization:** Qian Xu, Shuangbo Fan, Zhenping Zhao.

**Data curation:** Qian Xu, Shuangbo Fan, Ji Zheng, Yulin Wan, Rudong Tian, Zhenping Zhao.

**Formal analysis:** Qian Xu, Shuangbo Fan, Ji Zheng, Rudong Tian, Jia Xia.

**Funding acquisition:** Jia Xia.

**Methodology:** Liang Wang.

**Writing – original draft:** Qian Xu, Liang Wang, Ji Zheng, Yulin Wan, Rudong Tian, Zhenping Zhao.

**Writing – review & editing:** Qian Xu, Zhenping Zhao.

## Supplementary Material








