# Supplementary material for: Circulating/cerebrospinal T lymphocytes as indicators of clinical prognosis in intracerebral hemorrhage: A prospective study
Source: Medicine (Baltimore). 2024 Jul 19;103(29):e35827. doi: 10.1097/MD.0000000000035827 (PMC11398761; doi:10.1097/MD.0000000000035827)
Supplement: Supplementary file 1 [file medi-103-e35827-s001.docx]

**Circulating/Cerebrospinal T Lymphocytes as Indicators of Clinical Prognosis in Intracerebral Hemorrhage**

**Supplementry Information**

Table S1. The characteristics of circulating T lymphocytes in the study cohort

| **Indicators** | **Days after ICH** | **GCS>12**  **(n=19)** | **GCS≤12**  **(n=11)** | ***p* value** |
| --- | --- | --- | --- | --- |
| CD3^+^% | 1 | 62.7±13.1 | 62.5±4.34 | 0.94 |
|  | 7 | 65.0±11.2 | 61.6±14.4 | 0.51 |
|  | 14 | 73.5±5.45 | 67.3±8.90 | 0.056 |
| CD3^+^CD4^+^% | 1 | 43.3±10.8 | 43.8±6.33 | 0.88 |
|  | 7 | 39.6±8.61 | 38.5±10.1 | 0.75 |
|  | 14 | 39.7±5.38 | 35.7±6.91 | 0.12 |
| CD3^+^CD8^+^% | 1 | 15.1±5.05 | 13.6±2.27 | 0.30 |
|  | 7 | 21.1±7.04 | 18.8±4.62 | 0.30 |
|  | 14 | 28.1±6.37 | 26.0±10.1 | 0.56 |
| CD4^+^/CD8^+^ ratio | 1 | 3.08±0.82 | 3.34±0.99 | 0.46 |
|  | 7 | 2.04±0.64 | 2.09±0.49 | 0.8 |
|  | 14 | 1.50±0.44 | 1.54±0.55 | 0.82 |
| CD3^+^ count | 1 | 627.4±194.2 | 906.4±338.7 | **0.025** |
|  | 7 | 714.4±242.3 | 832.6±221.5 | 0.19 |
|  | 14 | 1421±325.7 | 1132±329.9 | **0.03** |
| CD3^+^CD4^+^ count | 1 | 433±151.2 | 627.1±225.7 | **0.022** |
|  | 7 | 431.3±149.7 | 522.4±167.8 | 0.15 |
|  | 14 | 765.6±195.4 | 611.1±210.9 | 0.061 |
| CD3+CD8+ count | 1 | 149.6±53.5 | 200.4±85.9 | 0.097 |
|  | 7 | 235.1±112.9 | 249.9±47.7 | 0.62 |
|  | 14 | 543.2±163.9 | 428.6±162.2 | 0.077 |

Note: ICH, intracerebral hemorrhage; GCS, Glasgow Coma Scale.
