# Supplementary material for: Circulating/cerebrospinal T lymphocytes as indicators of clinical prognosis in intracerebral hemorrhage: A prospective study
Source: Medicine (Baltimore). 2024 Jul 19;103(29):e35827. doi: 10.1097/MD.0000000000035827 (PMC11398761; doi:10.1097/MD.0000000000035827)
Supplement: Supplementary file 2 [file medi-103-e35827-s002.docx]

**Circulating/Cerebrospinal T Lymphocytes as Indicators of Clinical Prognosis in Intracerebral Hemorrhage**

Table S2. The AUC value of circulating T lymphocytes in the study cohort for GCS score

| **Indicators** | **Days after ICH** | **AUC** | **Standard error** | ***P*** | **95% CI** |
| --- | --- | --- | --- | --- | --- |
| CD3^+^% | 1 | 0.517 | 0.107 | 0.875 | 0.308 ~ 0.725 |
|  | 7 | 0.445 | 0.113 | 0.627 | 0.223 ~ 0.667 |
|  | 14 | 0.273 | 0.118 | 0.054 | 0.042 ~ 0.504 |
| CD3^+^CD4^+^% | 1 | 0.507 | 0.108 | 0.947 | 0.296 ~ 0.718 |
|  | 7 | 0.512 | 0.119 | 0.920 | 0.280 ~ 0.744 |
|  | 14 | 0.306 | 0.104 | 0.063 | 0.102 ~ 0.510 |
| CD3^+^CD8^+^% | 1 | 0.433 | 0.104 | 0.519 | 0.229 ~ 0.637 |
|  | 7 | 0.435 | 0.110 | 0.556 | 0.220 ~ 0.650 |
|  | 14 | 0.340 | 0.114 | 0.158 | 0.117 ~ 0.562 |
| CD4^+^/CD8^+^ ratio | 1 | 0.533 | 0.118 | 0.776 | 0.302 ~ 0.765 |
|  | 7 | 0.500 | 0.108 | 1.000 | 0.289 ~ 0.711 |
|  | 14 | 0.560 | 0.118 | 0.613 | 0.328 ~ 0.792 |
| CD3^+^ count | 1 | **0.766** | **0.098** | **0.007**** | **0.573 ~ 0.958** |
|  | 7 | 0.622 | 0.104 | 0.242 | 0.418 ~ 0.826 |
|  | 14 | 0.263 | 0.105 | 0.024* | 0.058 ~ 0.469 |
| CD3^+^CD4^+^ count | 1 | **0.744** | **0.103** | **0.018*** | **0.542 ~ 0.946** |
|  | 7 | 0.656 | 0.104 | 0.136 | 0.451 ~ 0.860 |
|  | 14 | 0.311 | 0.100 | 0.059 | 0.115 ~ 0.507 |
| CD3^+^CD8^+^ count | 1 | 0.675 | 0.107 | 0.102 | 0.465 ~ 0.884 |
|  | 7 | 0.610 | 0.104 | 0.289 | 0.406 ~ 0.814 |
|  | 14 | 0.318 | 0.110 | 0.097 | 0.103 ~ 0.533 |

Note: AUC, area under the curve; ICH, intracerebral hemorrhage; GCS, Glasgow Coma Scale; **P*<0.05, ***P*<0.01.
