# Supplementary material for: Circulating/cerebrospinal T lymphocytes as indicators of clinical prognosis in intracerebral hemorrhage: A prospective study
Source: Medicine (Baltimore). 2024 Jul 19;103(29):e35827. doi: 10.1097/MD.0000000000035827 (PMC11398761; doi:10.1097/MD.0000000000035827)
Supplement: Supplementary file 3 [file medi-103-e35827-s003.docx]

**Circulating/Cerebrospinal T Lymphocytes as Indicators of Clinical Prognosis in Intracerebral Hemorrhage**

Table S3. The characteristics of cerebrospinal T lymphocytes in the study cohort

| **Characteristic** | **Days after ICH** | **GCS>12**  **(n=12)** | **GCS≤12**  **(n=5)** | ***p* value** |
| --- | --- | --- | --- | --- |
| CD3^+^ % | 1 | 63.8±8.37 | 69.1±4.15 | 0.11 |
|  | 7 | 61.9±13.0 | 67.9±2.82 | 0.16 |
|  | 14 | 69.3±6.62 | 69.3±8.42 | 0.99 |
| CD3^+^CD4^+^% | 1 | 39.6±10.7 | 50.3±6.2 | **0.022** |
|  | 7 | 31.1±10.2 | 39.7±6.4 | 0.057 |
|  | 14 | 32.7±5.74 | 37.6±5.10 | 0.12 |
| CD3^+^CD8^+^% | 1 | 20.7±11.0 | 12.9±3.58 | **0.046** |
|  | 7 | 25.1±11.3 | 25.4±6.07 | 0.949 |
|  | 14 | 28.7±6.18 | 25.8±5.9 | 0.383 |
| CD4^+^/CD8^+^ ratio | 1 | 2.69±2.28 | 4.28±1.79 | 0.16 |
|  | 7 | 1.40±0.59 | 1.68±0.60 | 0.42 |
|  | 14 | 1.20±0.36 | 1.52±0.36 | 0.13 |
| CD3^+^ count | 1 | 573.3±393.6 | 1623±1109 | 0.10 |
|  | 7 | 1058±631.6 | 1621±1213 | 0.37 |
|  | 14 | 1659±1182 | 2367±1458 | 0.37 |
| CD3^+^CD4^+^ count | 1 | 360.7±263.4 | 1221.2±925.1 | 0.11 |
|  | 7 | 535.8±369.7 | 913.6±629.0 | 0.26 |
|  | 14 | 796.7±667.8 | 1323±868.2 | 0.27 |
| CD3^+^CD8^+^ count | 1 | 164.2±92.9 | 276.0±183.1 | 0.25 |
|  | 7 | 442.0±321.9 | 648.6±639.2 | 0.52 |
|  | 14 | 684.9±456.2 | 860.2±541.4 | 0.55 |

Note: ICH, intracerebral hemorrhage; GCS, Glasgow Coma Scale.
