# Supplementary material for: Circulating/cerebrospinal T lymphocytes as indicators of clinical prognosis in intracerebral hemorrhage: A prospective study
Source: Medicine (Baltimore). 2024 Jul 19;103(29):e35827. doi: 10.1097/MD.0000000000035827 (PMC11398761; doi:10.1097/MD.0000000000035827)
Supplement: Supplementary file 4 [file medi-103-e35827-s004.docx]

**Circulating/Cerebrospinal T Lymphocytes as Indicators of Clinical Prognosis in Intracerebral Hemorrhage**

Table S4. The AUC value of cerebrospinal T lymphocytes in the study cohort for GCS score

| **Indicators** | **Days after ICH** | **AUC** | **Standard error** | ***P*** | **95% CI** |
| --- | --- | --- | --- | --- | --- |
| CD3^+^% | 1 | 0.717 | 0.128 | 0.090 | 0.466 ~ 0.967 |
|  | 7 | 0.567 | 0.137 | 0.627 | 0.298 ~ 0.836 |
|  | 14 | 0.467 | 0.169 | 0.843 | 0.136 ~ 0.797 |
| CD3^+^CD4^+^% | 1 | **0.867** | **0.094** | **0.000**** | **0.683 ~ 1.050** |
|  | 7 | 0.750 | 0.141 | 0.076 | 0.474 ~ 1.026 |
|  | 14 | 0.700 | 0.132 | 0.129 | 0.442 ~ 0.958 |
| CD3^+^CD8^+^% | 1 | 0.267 | 0.128 | 0.069 | 0.015 ~ 0.518 |
|  | 7 | 0.617 | 0.142 | 0.410 | 0.339 ~ 0.894 |
|  | 14 | 0.317 | 0.156 | 0.241 | 0.010 ~ 0.623 |
| CD4^+^/CD8^+^ ratio | 1 | **0.917** | **0.080** | **0.000**** | **0.760 ~ 1.073** |
|  | 7 | 0.608 | 0.158 | 0.494 | 0.298 ~ 0.919 |
|  | 14 | 0.733 | 0.141 | 0.098 | 0.457 ~ 1.009 |
| CD3^+^ count | 1 | **0.867** | **0.096** | **0.000**** | **0.678 ~ 1.055** |
|  | 7 | 0.583 | 0.171 | 0.626 | 0.248 ~ 0.919 |
|  | 14 | 0.683 | 0.143 | 0.200 | 0.403 ~ 0.964 |
| CD3^+^CD4^+^ count | 1 | **0.867** | **0.096** | **0.000**** | **0.678 ~ 1.055** |
|  | 7 | 0.683 | 0.153 | 0.230 | 0.384 ~ 0.983 |
|  | 14 | 0.750 | 0.141 | 0.076 | 0.474 ~ 1.026 |
| CD3^+^CD8^+^ count | 1 | 0.700 | 0.148 | 0.176 | 0.410 ~ 0.990 |
|  | 7 | 0.600 | 0.158 | 0.526 | 0.291 ~ 0.909 |
|  | 14 | 0.617 | 0.150 | 0.436 | 0.323 ~ 0.910 |

Note: AUC, area under the curve; ICH, intracerebral hemorrhage; GCS, Glasgow Coma Scale; **P*<0.05, ***P*<0.01.
